# Multi-Targeted Neuroprotection by *Areca catechu* Against Cisplatin-Induced Neurotoxicity: Cellular, *Caenorhabditis elegans*, and Metabolomic Evidence

**DOI:** 10.3390/ijms27157004

**Published:** 2026-08-04

**Authors:** Kishore K. Kumaree, Clerance Su Yee Cheong, Kanika Verma, Kartina Nadyani, Nureesun Mahamud, Pornpimol Mahamad, Tewin Tencomnao, Anchalee Prasansuklab, James M. Brimson

**Affiliations:** 1 College of Public Health Sciences, Chulalongkorn University, Bangkok 10330, Thailand; kishoree.k3@gmail.com; 2 Center of Excellence on Natural Products for Neuroprotection and Anti-Ageing (Neur-Age NatChula), Chulalongkorn University, Bangkok 10330, Thailand; clerancecheong@gmail.com (C.S.Y.C.); kanika.honey.verma@gmail.com (K.V.); kartinanadyani@gmail.com (K.N.); tewin.t@chula.ac.th (T.T.); 3 Department of Clinical Chemistry, Faculty of Allied Health Sciences, Chulalongkorn University, Bangkok 10330, Thailand; 4 Program in Biotechnology, Faculty of Science, Chulalongkorn University, Pathum Wan, Bangkok 10330, Thailand; 5 The Halal Science Center, Chulalongkorn University, Bangkok 10330, Thailand; nureesun.hsc@gmail.com (N.M.); pornpimol.m.hsc@gmail.com (P.M.); 6 Center of Excellence in Metabolomics for Life Sciences, Chulalongkorn University, Bangkok 10330, Thailand; 7 Research, Innovation and International Affairs, Faculty of Allied Health Sciences, Chulalongkorn University, Bangkok 10330, Thailand

**Keywords:** platinum-based drugs, chemotherapy-associated neuropathy, areca nut, HT22, *C. elegans*, metabolomics

## Abstract

Cisplatin is an effective platinum-based chemotherapeutic agent used to treat a variety of cancers. However, its clinical utility is limited by dose-dependent neurotoxicity, yet no approved neuroprotective strategy currently exists. Our integrated cellular, metabolic, and in vivo approaches unraveled the neuroprotective potential of *Areca catechu* ethyl acetate extract (AC-EA) against cisplatin-induced neurotoxicity. Importantly, AC-EA did not reduce cisplatin-induced cytotoxicity in A549 lung cancer cells. In HT22 hippocampal neurons, AC-EA restored cell viability, suppressed reactive oxygen species generation, preserved mitochondrial-associated fluorescence, and attenuated phosphorylated histone H2AX (γH2AX)-marked DNA damage. AC-EA was associated with increased pNRF2 expression, consistent with activation of NRF2-dependent antioxidant signaling, suppressed inducible nitric oxide synthase iNOS (inducible nitric oxide synthase )-mediated neuroinflammation, and prevented the depletion of total AKT (protein kinase B) protein. Untargeted metabolomics and *Caenorhabditis elegans* survival assays were performed for mechanistic and in vivo validation. Metabolomics data showed restoration of several critical amino acids, including L-tyrosine and β-alanine, disrupted by cisplatin. Moreover, *C. elegans* studies confirmed in vivo activation of antioxidants via the SKN-1/GST-4 (glutathione S-transferase 4) pathway. While AC-EA shows neuroprotective potential, arecoline’s toxicity demands caution. To our knowledge, this is the first study to demonstrate the neuroprotective activity of *A. catechu* against cisplatin-induced neurotoxicity, laying the foundation for developing plant-derived adjunct therapies for chemotherapy-associated neuropathy.

## 1. Introduction

Cisplatin-based chemotherapy remains one of the most widely used and effective chemotherapeutic agents for treating a broad range of human malignant tumors, including ovarian, breast, bladder, and other solid tumors [1]. Despite its potent antitumor activity, the clinical application of cisplatin is severely limited by dose-limiting toxicities due to its non-selective mechanism of action. Among these toxicities, neurotoxicity, which manifests as both peripheral neuropathy and central nervous system dysfunction, is a major challenge to continuing treatment, often compromises patients’ quality of life, and frequently necessitates treatment discontinuation [2]. 

During cisplatin chemotherapy, neurotoxicity is a severe side effect that is noticeable in 30–50% of patients [3,4]. Chronic peripheral sensory neuropathy is common when the cumulative cisplatin dose is high. Neurotoxicity is a major reason for discontinuing cisplatin and for limiting its cumulative dose, which may diminish its chemotherapeutic efficacy [5]. Symptoms include uncomfortable distal paresthesias (tingling in the extremities), numbness, and Lhermitte’s sign (an electric-shock-like sensation during neck flexion), which are likely attributable to centripetal degeneration of the posterior columns [6]. These debilitating effects often require dose reduction, treatment delay, or premature cessation of chemotherapy, ultimately undermining good tumor control and potentially influencing patient prognosis.

The molecular mechanism of cisplatin-induced toxicity involves direct DNA binding, leading to DNA crosslinking, DNA damage, and genotoxic stress [7]. Platinum adducts accumulate in dorsal root ganglia (DRG) and neurons because these cells are not actively replicating. This indicates a significant correlation between cisplatin adduct levels and the severity of peripheral neurotoxicity [8]. Moreover, cisplatin exposure leads to the accumulation of reactive oxygen species (ROS), which triggers oxidative stress and cellular damage [9]. In neuronal cells, elevated ROS levels disrupt redox homeostasis and trigger apoptotic signaling [10]. Reduced levels of neurotrophic factors and mitochondrial damage further contribute to neuronal degeneration [11,12,13]. 

These molecular and physiological changes compromise therapeutic efficacy and severely worsen overall cancer prognosis. Therefore, developing effective neuroprotective therapies that preserve neuronal integrity without attenuating anticancer activity remains an urgent, unmet clinical need. Discovering therapeutic agents that mitigate cisplatin-induced neurotoxicity could enable patients to tolerate higher cumulative doses and complete the required chemotherapy regimens, ultimately improving survival outcomes [14]. However, there are no Food and Drug Administration (FDA) approved prophylactic agents to prevent cisplatin-induced neurotoxicity without compromising oncological efficacy [15].

In this context, natural products, particularly those rich in polyphenols and alkaloids, emerge as alternative neuroprotective agents due to their multitarget pharmacological activities [16,17,18]. *Areca catechu* L., commonly known as areca nut, is widely used in Asian countries and is one of the four major southern medicines in China [17]. Modern studies have identified various pharmacological effects of areca extract, including anti-inflammatory, antidepressant, antitumor, and antioxidant effects [19,20,21]. In addition, the anti-neuroinflammatory properties of *A. catechu* have been demonstrated in BV2 microglial cells [22], suggesting the ability to modulate neuronal inflammatory pathways implicated in chemotherapy-induced neurotoxicity. Despite its promising pharmacological profile, the potential of *A. catechu* extract to counteract cisplatin-induced neurotoxicity has not been systematically explored, representing a significant gap in the literature. Although cisplatin neurotoxicity is clinically dominated by peripheral sensory neuropathy, HT22 cells are of hippocampal origin. Therefore, the present model was used to investigate the general neuronal stress response.

Therefore, this study aims to examine the protective effects of *A. catechu* extract against cisplatin-induced neurotoxicity in HT22 cells and to investigate the underlying molecular and metabolic mechanisms using integrated cellular, metabolic, and *Caenorhabditis elegans* evidence.

## 2. Results

### 2.1. Phytocompounds Analysis of AC-EA

To identify the bioactive constituents of AC-EA, the crude extract was analyzed by liquid chromatography- tandem mass spectrometry (LC-MS/MS). Several putative phytocompounds were identified based on accurate MS1 masses and MS/MS spectral matches in the METLIN and MetFrag databases. A major early eluting peak at RT 1.8 min corresponded to arecoline (*m*/*z* 156.101), a well-known bioactive alkaloid of *A. catechu*. A detailed list of several other identified compounds, including their retention times and molecular formulas, is summarized in Table 1 and Supplementary Figure S1. The putative phytocompounds indicate that the AC-EA fraction is enriched in alkaloids, polyphenols, terpenes, and fatty acids, which may contribute to its observed biological activities. These represent putative identifications, as they are based on MS/MS spectral library matching without confirmation via purified analytical standards.

### 2.2. Cytotoxicity Profile and Neuroprotective Effects of AC-EA in Cisplatin-Treated Cells

**Figure 1:**
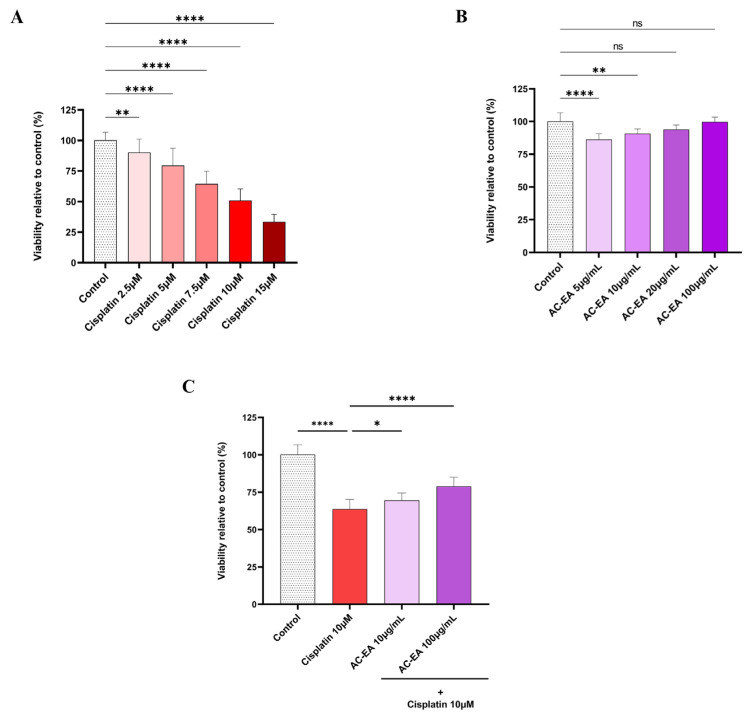
(**A**) Cytotoxic effect of cisplatin alone, (**B**) Intrinsic toxicity of AC-EA alone, and (**C**) AC-EA co-treatment with cisplatin on HT22 cell viability. Cell viability was assessed using the MTT assay and expressed as a percentage relative to the control group. Data represent the mean ± SD (*n* = 3). Statistical significance was determined using one-way ANOVA with Dunnett’s post hoc test against the control for graphs (**A**,**B**). For (**C**), significance was calculated relative to cisplatin. (* *p* < 0.05, ** *p* < 0.01, **** *p* < 0.0001, ns: not significant).

To establish our in vitro neurotoxicity model, HT22 cells were exposed to increasing concentrations of cisplatin (2.5–15 µM) for 24 h. Cisplatin treatment resulted in a dose-dependent reduction in cell viability compared with the control group (Figure 1A). The 10 µM cisplatin dose was selected because it produced a cytotoxic effect, reducing HT22 cell viability to 50–60%. The cytotoxicity of the AC-EA extract was assessed to ensure its efficacy. HT22 cells were treated with AC-EA alone (5–100 µg/mL), which showed no significant toxicity, maintaining cell viability above 85% across all tested concentrations (Figure 1B).

The neuroprotective potential of the extract is shown in Figure 1C. Cells were co-treated with cisplatin (10 µM) and AC-EA (10 µg/mL or 100 µg/mL). Co-treatment with AC-EA significantly improved cell viability in HT22 cells compared with the cisplatin-treated group alone. AC-EA co-treatment showed a dose-dependent protective effect, with the 100 µg/mL dose restoring cell viability to 78.9% ± 5.8 (*p* < 0.0001 vs. the cisplatin group).

**Figure 2:**
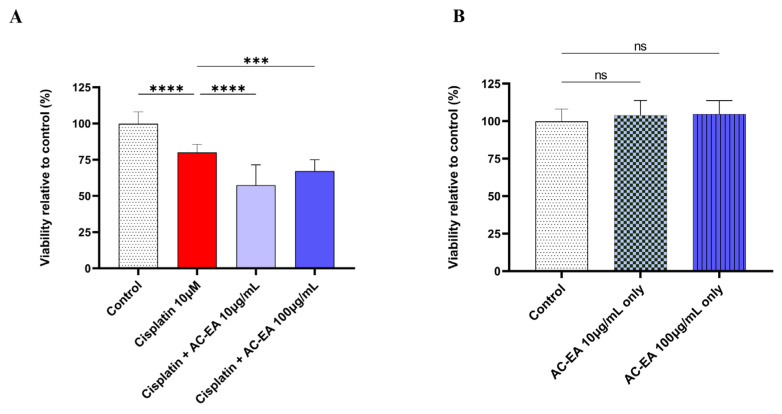
Evaluation of AC-EA on the chemotherapeutic efficacy of cisplatin in A549 lung cancer cells. (**A**) Cell viability was assessed using the MTT assay following 24 h of co-treatment with cisplatin (10 µM) and AC-EA (10 and 100 µg/mL). (**B**) Effects of AC-EA alone on A549 cell viability. The data are expressed as a percentage relative to the control group (set at 100%). Data represent the mean ± SD (*n* = 3). Statistical significance was determined using one-way ANOVA followed by Dunnett’s post hoc test against cisplatin in (**A**) and against the control in (**B**). (*** *p* < 0.001, **** *p* < 0.0001, ns: not significant).

Furthermore, a critical requirement of this assessment is to determine whether the extract interferes with the chemotherapeutic efficacy of cisplatin. To address this, we assessed the viability of the A549 lung cancer cell line after co-treatment with AC-EA and cisplatin. As shown in Figure 2A, AC-EA did not compromise the chemotherapeutic efficacy of cisplatin and did not confer protection to the cancer cells, as it did to HT22 neuronal cells. AC-EA alone at 10 and 100 µg/mL did not significantly affect A549 cell viability compared with the control. These findings indicate that AC-EA did not attenuate cisplatin-induced cytotoxicity in A549 cells under the tested conditions (*p* < 0.0001 and *p* < 0.001, respectively, Dunnett’s post hoc test).

### 2.3. Effects of AC-EA on Intracellular ROS Generation and Mitochondrial Status

**Figure 3:**
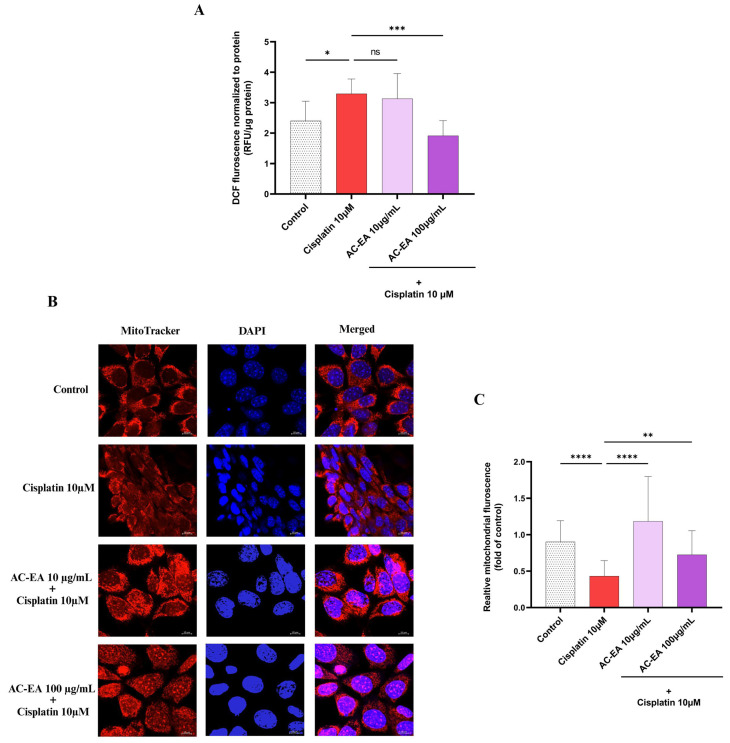
The effect of AC-EA on oxidative stress and mitochondrial-associated fluorescence in cisplatin-treated HT22 cells. (**A**) Intracellular ROS generation was quantified using the H_2_DCFDA fluorescent probe. Background-corrected DCF fluorescence was normalized to total cellular protein and expressed as RFU/µg protein. (**B**) Representative confocal images showing MitoTracker-stained mitochondria and DAPI-stained nuclei in control, cisplatin-treated, and AC-EA co-treated cells. (**C**) Quantification of relative mitochondrial fluorescence intensity normalized to control. Cells were stained with MitoTracker Red (red) to visualize mitochondria and DAPI (blue) to label nuclei. Scale bar = 10 µm. Data represent the mean ± SD (*n* = 3). Statistical significance was determined using one-way ANOVA followed by Dunnett’s post hoc test against the cisplatin group (* *p* < 0.05, ** *p* < 0.01, *** *p* < 0.001, **** *p* < 0.0001, ns: not significant).

Furthermore, to determine whether the neuroprotective effect of AC-EA is mediated by regulation of oxidative stress, intracellular ROS levels were measured using the H_2_DCFDA assay; raw fluorescence values were normalized to total cellular protein content. As shown in Figure 3A, the cisplatin (10 µM) group showed a significant increase in ROS generation compared to the control. However, co-treatment with AC-EA at 10 µg/mL did not significantly reduce ROS production. Interestingly, the 100 µg/mL dose was the most effective, showing a significant reduction in ROS levels of approximately 40% compared to cisplatin treatment (*p* < 0.001).

Since mitochondria are the primary source of intracellular ROS during cisplatin toxicity, we next assessed mitochondrial-associated fluorescence using MitoTracker staining (Figure 3B,C). Cisplatin treatment resulted in a severe reduction in mitochondrial fluorescence intensity compared with control-treated cells, indicating mitochondrial dysfunction. In contrast, co-treatment with AC-EA significantly reversed this effect. Among the tested co-treatments, AC-EA at 10 µg/mL produced the highest MitoTracker fluorescence, whereas the 100 µg/mL dose showed stronger effects in several other experiments. These findings suggest that AC-EA protects against cisplatin-induced neurotoxicity by preventing mitochondrial dysfunction and the subsequent ROS generation in neuronal cells.

### 2.4. AC-EA Attenuates Cisplatin-Induced DNA Damage

Continuous exposure of cells to oxidants damages DNA, leading to intrastrand crosslinks and fatal double-strand breaks. This is a major mechanism of cisplatin [23]. To determine whether AC-EA alleviates genotoxic stress, we examined phosphorylation of histone H2AX (γH2AX), an established marker of double-strand DNA breaks (DSBs) generated by *Ser*139 phosphorylation in response to DNA damage [24].

**Figure 4:**
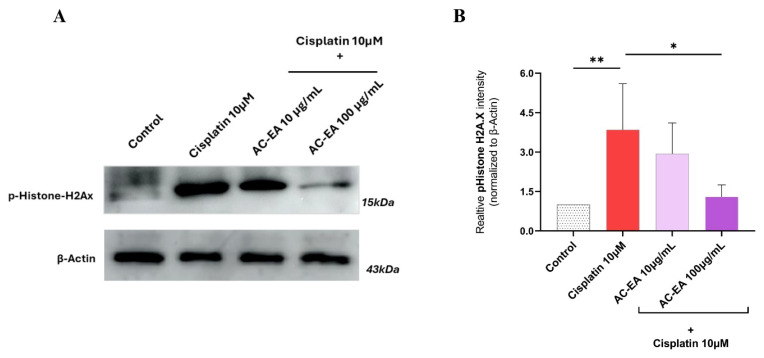
Effect of AC-EA on cisplatin-induced DNA damage. Western blot analysis was performed to assess expression of the DNA double-strand break marker γH2AX (p-Histone-H2A.X). (**A**) Representative immunoblots of γH2AX protein expression across all treated groups in HT22 cells. (**B**) Quantification of γH2AX band intensity normalized to β-actin. Data are presented as mean ± SD (*n* = 4). Statistical significance was determined using one-way ANOVA with Dunnett’s post hoc test against the cisplatin group (* *p* < 0.05, ** *p* < 0.01).

Exposure to cisplatin (10 µM) led to a marked accumulation of γH2AX compared with control cells, confirming substantial intracellular damage (Figure 4A). However, co-treatment with AC-EA attenuated this response in a dose-dependent manner (Figure 4B). The 10 µg/mL dose produced a non-significant reduction, whereas the 100 µg/mL dose significantly suppressed γH2AX accumulation compared with cisplatin alone (*p* < 0.05). These results indicate that AC-EA confers measurable protection against cisplatin-induced DNA damage.

### 2.5. AC-EA Modulates Inflammatory, Antioxidant and AKT-Associated Protein Expression

**Figure 5:**
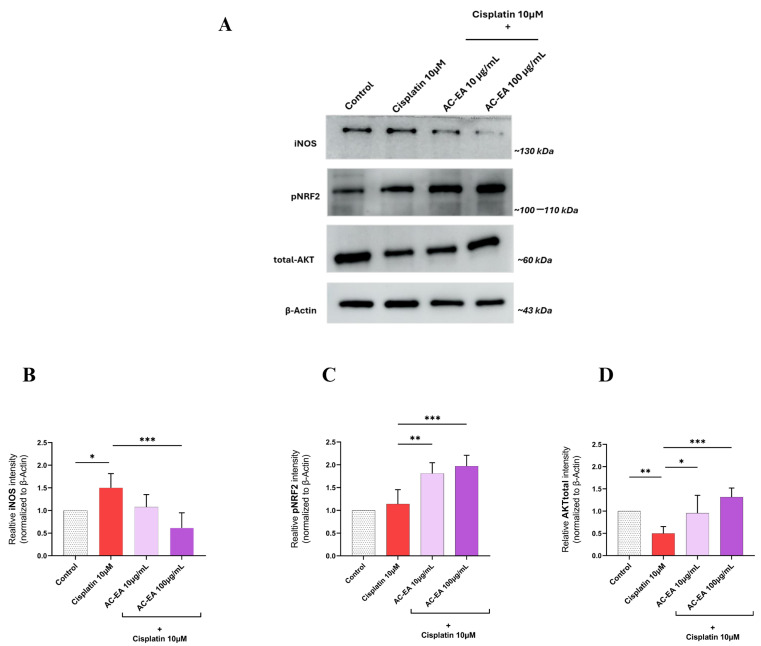
AC-EA co-treatment could modulate inflammatory and antioxidant signaling pathways. (**A**) Representative immunoblots showing protein expression of iNOS, pNRF2, and total AKT in cisplatin-treated HT22 cells. β-actin served as an internal loading control. (**B**–**D**) Quantification of iNOS, pNRF2, and total AKT band intensities normalized to β-actin. Data are presented as mean ± SD (*n* = 4). Statistical significance was assessed using one-way ANOVA with Dunnett’s post hoc test versus the cisplatin group (* *p* < 0.05, ** *p* < 0.01, *** *p* < 0.001).

After establishing the protective effect of AC-EA against cisplatin-induced DNA damage, we next examined the expression of key inflammatory (iNOS), antioxidant (pNRF2), and survival (total AKT) proteins (Figure 5A). Cisplatin treatment (10 µM) induced significant inflammatory stress, indicated by upregulation of iNOS (*p* < 0.05), while simultaneously suppressing total AKT protein levels (*p* < 0.01). However, co-treatment with AC-EA reversed these effects in a dose-dependent manner. The 100 µg/mL dose significantly attenuated iNOS expression (*p* < 0.001) (Figure 5B) and strongly upregulated the expression of the antioxidant regulator pNRF2 compared to the cisplatin-only group (*p* < 0.001) (Figure 5C). The increase in pNRF2 expression was associated with restoration of total AKT expression to control levels (*p* < 0.001) (Figure 5D), consistent with enhanced antioxidant signaling and the preservation of cellular AKT protein pools. Notably, the NRF2 bands were observed at approximately 100–110 kDa despite its predicted molecular mass of approximately 68 kDa, demonstrating the well-known anomalous electrophoretic migration of NRF2.

### 2.6. Docking Analysis

**Figure 6:**
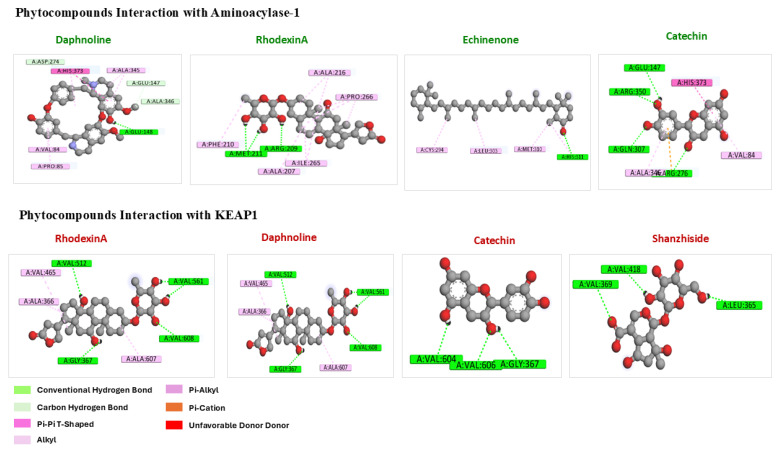
Molecular docking interactions of selected phytocompounds identified in AC-EA with KEAP1 and Aminoacylase-1 proteins.

Molecular docking analysis was conducted as a hypothesis-generating approach to explore potential interactions between AC-EA phytocompounds and Kelch-like ECH-associated protein 1 (KEAP1) or aminoacylase-1. Rhodexin A showed the most favorable predicted binding energy for KEAP1 (−10.793 kcal/mol), followed by Daphnoline (−9.964 kcal/mol), catechin (−9.201 kcal/mol), and Shanzhiside (−8.158 kcal/mol). In contrast, docking results for aminoacylase-1 indicated Daphnoline as the most potent compound (−7.911 kcal/mol), with Rhodexin A (−7.629 kcal/mol), Echinenone (−6.951 kcal/mol), and catechin (−6.612 kcal/mol) also showing significantly favorable predicted docking energies within key regions of the enzyme. Notably, Daphnoline, Rhodexin A, and catechin exhibited the most favorable predicted docking scores for both KEAP1 and aminoacylase-1, suggesting they may interact with proteins involved in multiple metabolic pathways. However, these results are computational predictions and do not confirm direct target engagement. Overall, these findings indicate that AC-EA-derived phytochemicals may have diverse metabolic effects by simultaneously interacting with key proteins involved in cellular redox regulation and amino acid metabolism. The molecular interaction analysis of the docked phytocompounds revealed multiple stabilizing interactions within the active sites of the target proteins. These interactions highlight the potential of the phytocompounds to form stable, specific interactions with key amino acid residues, reinforcing their role as promising bioactive agents (Figure 6; Supplementary Table S1).

### 2.7. AC-EA Modulates Cisplatin-Induced Metabolic Alterations Induced by Cisplatin in HT22 Cells

**Figure 7:**
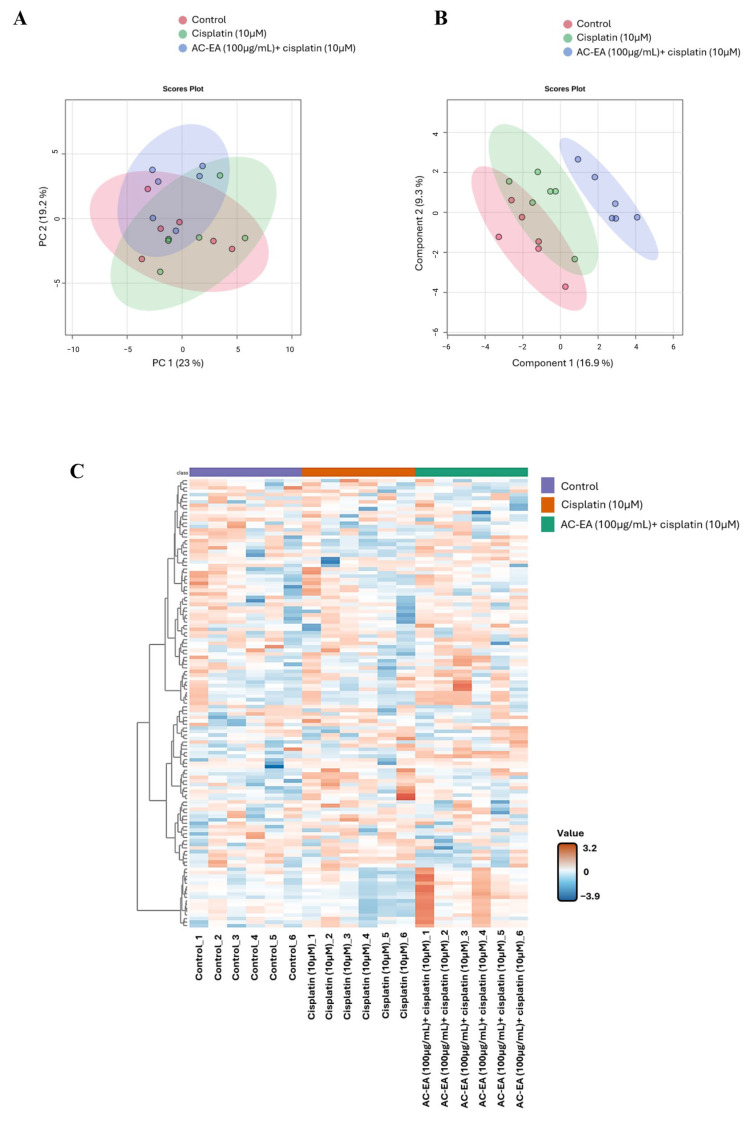
Multivariate statistical analysis of metabolic profiles in HT22 cells. (**A**) Unsupervised Principal Component Analysis (PCA) scores plot for control, cisplatin (10 µM), and AC-EA (100 µg/mL) plus cisplatin groups (PC1: 23%, PC2: 19.2%). (**B**) Supervised Partial Least Squares-Discriminant Analysis (PLS-DA) scores plot. (**C**) Hierarchical clustering heatmap of global metabolite abundance. Rows represent individual metabolic features, and columns represent biological replicates. The color scale indicates relative abundance (Red: high; Blue: low).

The molecular mechanistic analysis was further conducted using a metabolomics study in MetaboAnalyst 6.0 (Supplementary Table S2). Initially, unsupervised Principal Component Analysis (PCA) was performed to assess variation among the control, cisplatin (10 µM), and AC-EA (100 µg/mL) groups. The PCA score plot showed clear separation of metabolic profiles among the groups, with the first two principal components accounting for 42.2% of the total variance (Figure 7A).

To further clarify differences among these metabolic profiles and maximize group separation, we used the supervised Partial Least Squares-Discriminant Analysis (PLS-DA) model. The PLS-DA results showed a significant separation between the cisplatin and AC-EA groups (Figure 7B). PLS-DA cross-validation indicated moderate predictive ability (Q^2^ = 0.35) and high explanatory power (R^2^ > 0.9). Permutation testing confirmed that the separation was statistically significant (PERMANOVA, *p* = 0.02), supporting reliable group separation and indicating that the AC-EA intervention effectively shifts the metabolic phenotype away from the cisplatin-induced state. While the relatively modest Q^2^ value reflects the inherent variability of untargeted metabolomics data, the separation was not attributed to overfitting. 

The heatmap shows that cisplatin-treated (10 µM) cells exhibited a distinct pattern with broader shifts in metabolite levels, indicated by both increased (red) and decreased (blue) intensities, suggesting that cisplatin caused extensive metabolic alterations (Figure 7C). In contrast, AC-EA (100 µg/mL) co-treatment samples displayed several metabolites with reduced deviation from control levels.

To understand the metabolic differences between the toxic and treatment groups, we conducted additional multivariate statistical analyses focused solely on the cisplatin (10 µM) and AC-EA (100 µg/mL) plus cisplatin groups. Initially, PCA revealed a distinct change in the metabolic profile between the two groups (Supplementary Figure S2A).

To further elucidate metabolic differences, we used the OPLS-DA model to analyze data from the cisplatin and AC-EA groups (Supplementary Table S3). The two groups showed distinct separation, indicating significant changes in the cellular metabolite profile after AC-EA administration (Supplementary Figure S2B). The model demonstrated good accuracy and predictive ability (Q^2^; *p* = 0.003), confirming that the differences between the groups were statistically significant and not due to overfitting.

### 2.8. Exploratory Pathway Analysis and Discriminatory Metabolites Associated with the Neuroprotective Effect of AC-EA

**Figure 8:**
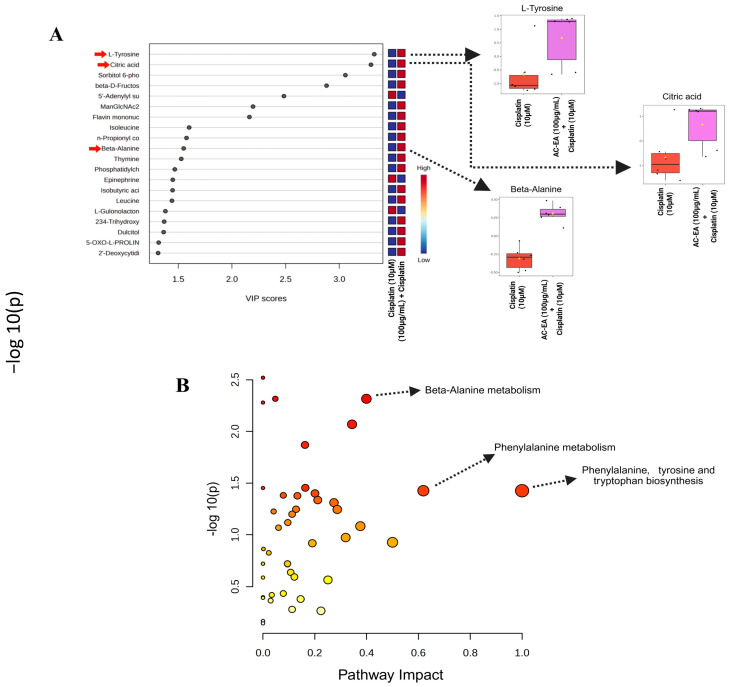
Discriminatory metabolites and exploratory pathway analysis associated with AC-EA co-treatment. (**A**) Variable Importance in Projection (VIP) scores plot of the top differential metabolites (VIP > 1.0). Boxplots show the relative abundance of L-Tyrosine, Citric acid, and β-Alanine, indicating their relative abundance distribution across groups (horizontal line: median). (**B**) Pathway topology analysis of HT22 cells treated with cisplatin (10 µM) alone and cisplatin (10 µM) plus AC-EA (100 µg/mL). The x-axis represents pathway impact, and the y-axis represents significance (−log(*p*)). Node color and radius correspond to the *p*-value and pathway impact, respectively. Red arrows in panel (**A**) indicate the three selected discriminatory metabolites L-tyrosine, citric acid, and β-alanine shown in the corresponding boxplots.

The identified metabolites were further evaluated using VIP scores to assess their contribution to separation between the Cisplatin and AC-EA-treated groups. Several metabolites had VIP values greater than 1.0, indicating their contribution to group discrimination. Among the top metabolites were L-tyrosine, citric acid, and β-alanine (Figure 8A). These metabolites were therefore selected for further interpretation as distinct metabolites associated with AC-EA co-treatment. 

Pathway analysis using MetaboAnalyst 6.0 identified several nominally enriched pathways based on uncorrected *p*-values (Figure 8B; Table 2). Phenylalanine, tyrosine, and tryptophan biosynthesis had the highest pathway impact score of 1.0. The top ten pathways, ranked by nominal *p*-value, are presented in the Supplementary Data (Supplementary Table S4). These findings indicate that AC-EA’s neuroprotective effects may be mediated by regulating amino acid biosynthesis and energy-related metabolic pathways disrupted by cisplatin.

**Figure 9:**
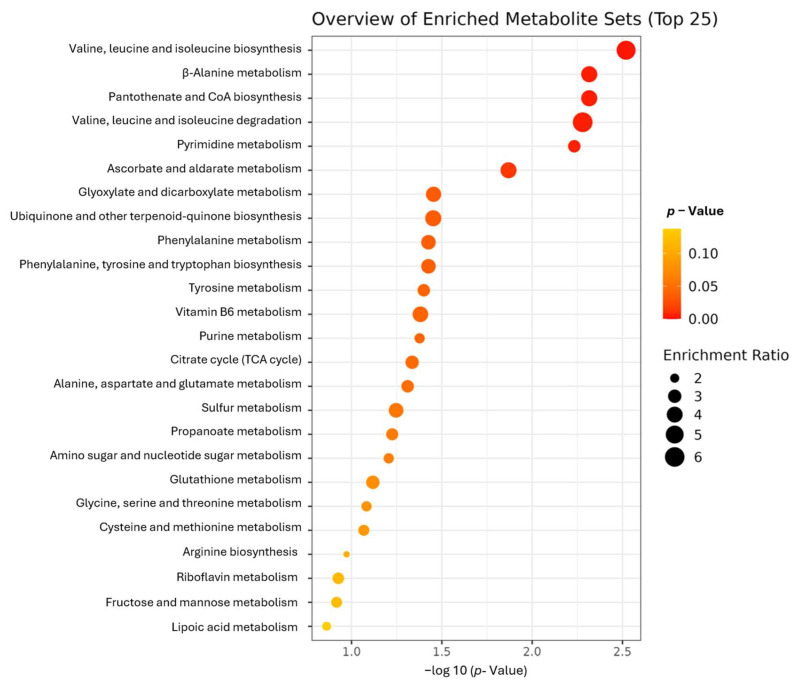
The Enrichment analysis displays the top 25 enriched metabolic pathways identified based on the most altered metabolites between cisplatin-alone and AC-EA-co-treated groups. The x-axis represents pathway significance expressed as −log10 (*p*-value), while the size of the dot highlights the enrichment ratio (i.e., the number of matched metabolites relative to the total number of metabolites in the respective pathway). The color of the dot corresponds to the statistical significance, with darker red indicating lower *p*-values.

Metabolite enrichment was performed to explore the broader metabolic context of the observed changes. The analysis revealed nominal associations with the upregulation of amino acid pathways, including branched-chain amino acid synthesis and degradation, β-alanine metabolism, and phenylalanine-tyrosine-tryptophan metabolism (Figure 9). Moreover, pathways related to mitochondrial energy production and oxidative stress regulation, such as the citric acid cycle (TCA), pantothenate and CoA synthesis, and vitamin B6 metabolism, were also enriched. These enrichment results provide a possible metabolic context for the observed metabolite changes. Accordingly, the individual changes in L-tyrosine, citric acid, and β-alanine provide the most direct evidence of metabolic alterations associated with AC-EA co-treatment.

### 2.9. AC-EA Enhances Stress Resistance and Activates Antioxidant Signaling in C. elegans

**Figure 10:**
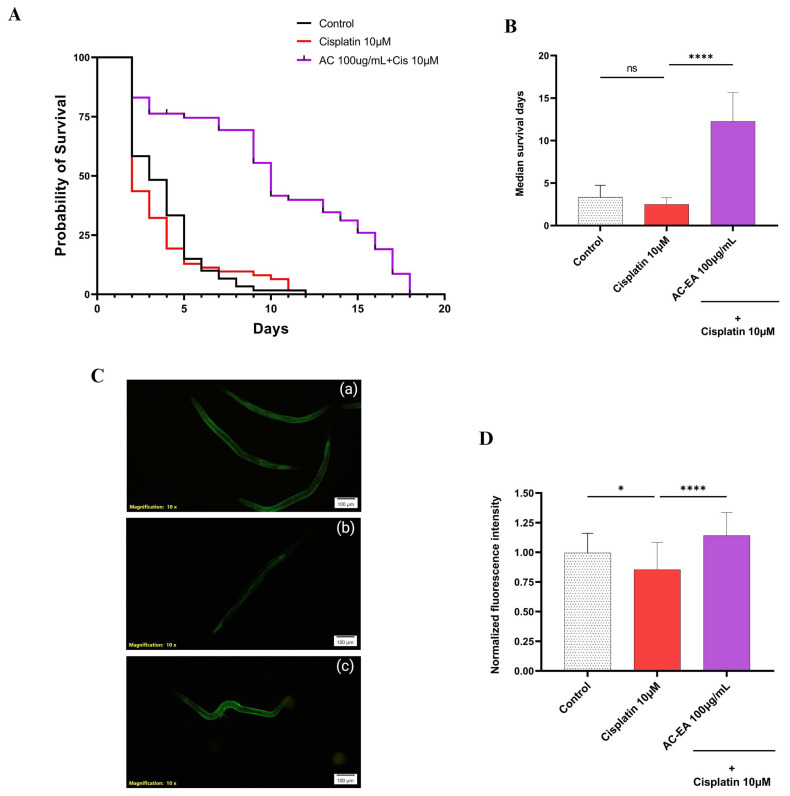
AC-EA improves survival and antioxidant gene expression in *C. elegans* under stress. (**A**) Kaplan–Meier survival curves of nematodes exposed to acute oxidative stress (50 µM Juglone). The *C. elegans* survival curve reflects survival following a transient oxidative challenge via a single juglone exposure. To establish treatments, worms were pre-treated for 24 h with vehicle (control), Cisplatin (10 µM) alone, or an AC-EA (100 µg/mL) + Cisplatin (10 µM) co-treatment, followed by uniform exposure of all groups to 50 µM juglone. AC-EA (100 µg/mL) significantly extended survival compared to the Cisplatin and Control groups (*p* < 0.0001, Log-rank test). (**B**) Median survival days (*n* = 6), (**C**) Representative fluorescence images of *gst-4::GFP* transgenic worms (*n* = 3): (**a**) Control, (**b**) Cisplatin 10 µM alone, and (**c**) AC-EA 100 µg/mL plus Cisplatin 10 µM. (**D**) Quantification of mean fluorescence intensity. Data are presented as mean ± SD (*n* = 3). Statistical significance was determined using one-way ANOVA followed by Dunnett’s post hoc test against the cisplatin group (* *p* < 0.05, **** *p* < 0.0001, ns: not significant).

To further validate the neuroprotective potential of AC-EA in vivo, we used the *C. elegans* model. We performed a survival assay under acute oxidative stress induced by juglone (50 µM). Because juglone was administered as a single exposure without replenishment, the survival curve here represents long-term survival following a transient oxidative challenge. As shown in Figure 10A, exposure to cisplatin under oxidative stress significantly reduced nematode survival. However, co-treatment with AC-EA (AC-EA 100 µg/mL plus cisplatin 10 µM) extended the median survival time from 2.5 days (cisplatin 10 µM group) to 12 days (*p* < 0.0001), significantly shifting the survival curve to the right.

To determine whether this protection was mediated by the SKN-1/NRF2 pathway, we quantified GST-4 expression in the transgenic strain CL2166 (*gst-4*::GFP). Co-treatment with AC-EA significantly increased GFP intensity (Figure 10B). Image intensity quantification showed that AC-EA treatment increased GST-4 expression by ~1.4-fold compared with the cisplatin group (*p* < 0.001).

## 3. Discussion

For decades, chemotherapy-induced neurotoxicity has been extensively investigated; however, strategies to manage it have received little attention. For patients receiving cisplatin, peripheral neuropathy is not merely a side effect; it forces a lifestyle choice between tolerating neurological deterioration and accepting a reduction in the very treatment. Nevertheless, despite decades of research into cisplatin’s mechanism of action, no FDA-approved drug is available to prevent this toxicity, which ultimately compromises cancer treatment. 

This study demonstrated that ethyl acetate extract of *A. catechu* (AC-EA) exerts neuroprotective effects against cisplatin-induced neurotoxicity in vitro without altering cisplatin toxicity in cancer cells. Preserving anticancer activity is crucial for any adjunct therapy intended to reduce chemotherapy-induced neurotoxicity. Although AC-EA protected neuronal cells from cisplatin-induced toxicity, it did not reduce cisplatin-mediated cytotoxicity in A549 lung cancer cells. This selective activity is particularly important for developing adjunct therapies designed to mitigate chemotherapy-induced neurotoxicity, as preserving anticancer efficacy is a fundamental requirement for clinical translation.

Our findings show that AC-EA protects cells by preventing oxidative stress- and mitochondrial dysfunction-induced cellular damage. Moreover, in this study, we adopted a systems biology approach, integrating metabolomics with cellular and molecular analyses to comprehensively characterize the key metabolic pathways modulated by AC-EA that rescue HT22 cells from cisplatin-induced neurotoxicity.

Herbal treatments, including supplementation forms or total crude extracts, are widely used as complementary therapies, particularly among patients undergoing chemotherapy [25,26,27]. Although *A. catechu* and its decoctions have been extensively investigated for their therapeutic potential in neuroinflammation and cognitive disorders [22,28,29], the plant’s efficacy against chemotherapy-induced neurotoxicity remains unexplored. To the best of our knowledge, this is the first study to investigate the neuroprotective potential of *A. catechu* in the context of cisplatin-induced neuronal damage.

To elucidate the basis for this potential neuroprotection, we characterized the bioactive constituents of AC-EA using LC-MS/MS. We identified a diverse array of bioactive compounds (Table 1), including arecoline, catechin, and the amino acid derivative N-Acetyl-L-phenylalanine. The neuroprotective activity of herbal extracts is often attributed to synergistic effects among multiple metabolites rather than to a single compound. Catechin is a well-documented antioxidant that acts as a direct ROS scavenger by modulating the NRF2 pathway [30,31], whereas arecoline, the major constituent of areca nut, has been reported to exert neuroprotective effects by alleviating neuroinflammation and reducing the pro-inflammatory cytokine IL-1β [32]. However, chronic exposure to arecoline has also been associated with adverse toxicological effects [33,34]. The presence of these metabolites explains the observed biological outcome and supports the notion that AC-EA exerts its effects through coordinated, multitarget neuroprotection.

Cisplatin-induced cellular toxicity was confirmed by an MTT assay (Figure 1). An important consideration for any proposed adjunct neuroprotective agent used during chemotherapy is whether it compromises anticancer efficacy. In the present study, AC-EA does not attenuate cytotoxicity in cancer cells (Figure 2), suggesting that its protective effects may be more selective for neuronal cells rather than broadly reducing cisplatin toxicity. This finding strengthens the translational relevance of AC-EA as a potential adjunct strategy to reduce chemotherapy-associated neurotoxicity. Nevertheless, further studies across additional cancer types and in combination with clinically relevant cisplatin dosing schedules are required before therapeutic application can be considered.

In our study, we observed that the AC-EA extract could help scavenge cisplatin-induced cytosolic free radical formation. Normalizing H_2_DCFDA fluorescence to total protein content showed that cisplatin significantly increased ROS generation (Figure 3A), confirming that ROS is a major contributor to the side effects observed in neuronal cells. Mechanistically, the restoration of redox homeostasis may be a principal mechanism of AC-EA-mediated neuroprotection. This effect may be related to the combined antioxidant action of the putative phytocompounds identified in AC-EA (Table 1), such as polyphenols, which may reduce oxidative stress through direct radical-scavenging activity. This is consistent with a recent study demonstrating that polyphenolic compounds effectively mitigated cisplatin-induced cellular toxicity by directly scavenging reactive oxygen species [35]. Overall, these findings suggest that AC-EA protects neurons by restoring intracellular redox balance. 

Oxidative stress and mitochondrial dysfunction are interdependent and intrinsically linked in the pathogenesis of neurotoxicity, as oxidative stress can cause mitochondrial damage, which in turn intensifies ROS generation, establishing a damaging loop of cellular injury [36]. Our results show that cisplatin treatment significantly reduced MitoTracker fluorescence, indicating disruption of mitochondrial redox status and membrane potential-associated fluorescence activity (Figure 3B). In contrast, AC-EA co-treatment restored mitochondrial fluorescence, suggesting that AC-EA may help preserve mitochondrial homeostasis during cisplatin exposure. This effect was accompanied by a parallel reduction in intracellular ROS levels (Figure 3A), indicating that AC-EA may interrupt the interaction between oxidative stress and mitochondrial dysfunction. The lower concentration produced the greatest MitoTracker fluorescence, whereas the higher dose showed better efficacy in other cellular protection assays. The putative phytocompounds present in AC-EA may contribute to this effect by reducing oxidative burden and limiting secondary mitochondrial damage. This interpretation is consistent with recent evidence indicating that reducing ROS accumulation and preserving mitochondrial membrane potential can attenuate chemotherapy-induced neuronal damage [37].

Cisplatin causes neurotoxicity by forming DNA adducts; meanwhile, cisplatin-induced oxidative stress further contributes to this genotoxicity by activating DNA damage responses, leading to DSBs and impairing the cellular repair system. To assess these phenomena, we examined γH2AX expression, a marker of the DNA-damage response associated with DSBs. The AC-EA co-treatment group shows a significant reduction in γH2AX phosphorylation in cisplatin-exposed HT22 cells (Figure 4), indicating attenuation of the cisplatin-induced DNA damage response in neuronal cells. This protective effect could be attributed to the downstream effects of mitochondrial protection and ROS scavenging observed in our study. Consistent with this interpretation, a previous study has shown that phytochemical-rich extracts attenuated cisplatin-induced DNA damage by preventing γH2AX phosphorylation, further emphasizing the therapeutic potential of plant metabolites in preserving genomic integrity [38].

To better understand the signaling mechanisms underlying AC-EA’s protective effects, we analyzed redox-responsive signaling proteins. NRF2 is a key regulator of cellular antioxidant defense, and its activation is linked to neuroprotection and an anti-inflammatory response. It has been reported to confer protection against oxidative stress [39]. AC-EA co-treatment significantly upregulated phosphorylated NRF2 expression (Figure 5), suggesting enhanced NRF2-associated antioxidant signaling. This is consistent with previous findings showing that flavonoid-rich herbal extracts could alleviate neuronal injury by activating the PI3K/Akt/Nrf2 signaling pathways [40,41].

AC-EA also restored total AKT expression in cisplatin-treated cells (Figure 5). Mechanistically, activated AKT can promote neuronal survival and facilitate the nuclear translocation of NRF2, which subsequently activates the endogenous antioxidant response [30]. Preservation of total AKT abundance may indicate maintenance of cellular survival capacity during cisplatin exposure. Nevertheless, total AKT expression alone does not demonstrate AKT signaling activation.

Furthermore, AC-EA treatment simultaneously suppressed cisplatin-induced iNOS expression. Overactivation of iNOS during oxidative stress can increase nitric oxide production, thereby contributing to neuronal injury. Earlier studies have confirmed the direct involvement of dysregulated iNOS activity in the development of several neurodegenerative processes and neuroinflammatory conditions [42,43]. Hence, the reduction in iNOS expression indicates that AC-EA may attenuate cisplatin-associated inflammatory and nitrosative stress. Together with the increase in phospho-NRF2 and the reduction in iNOS expression, these findings suggest that AC-EA triggers a coordinated antioxidant and anti-inflammatory response. To validate whether this cellular protection translates into in vivo organismal resilience, we used the *C. elegans* model.

Because cisplatin-induced neurotoxicity is intrinsically linked to ROS accumulation, we evaluated whether AC-EA could improve *C. elegans* resistance to a juglone-induced oxidative stress model. Nematodes treated with cisplatin alone showed reduced survival, whereas AC-EA co-treatment significantly shifted the survival curve and prolonged worms’ lifespan (Figure 10A). This improvement was accompanied by systemic activation of endogenous antioxidant defenses, as confirmed by a transgenic *gst-4*::GFP strain (Figure 10C,D), supporting enhanced expression of enzymes associated with SKN-1 signaling (the ortholog of mammalian NRF2). These findings are consistent with increased phosphorylated NRF2 expression and reduced oxidant-associated fluorescence observed in HT22 cells. Because an AC-EA-only group was not included, the prolonged survival cannot be exclusively attributed to protection against cisplatin; it may partly reflect a broader increase in stress resistance. 

Neurons are vulnerable to oxidative stress, and at the cellular level, several metabolites can act as stress inducers. Using untargeted metabolomics, we aimed to understand the effects of AC-EA co-treatment on neuronal cells. We sought to determine whether maintaining mitochondrial integrity and cellular survival could also preserve metabolic homeostasis. Recent studies have emphasized that maintaining metabolic stability is important for neuronal resilience against stress-induced injury [44]. Among the discriminatory metabolites, cisplatin treatment reduced intracellular L-tyrosine (Figure 8A), a key precursor of catecholamine neurotransmitters [45]; hence, its depletion may reflect disruption of neuronal amino acid homeostasis during cisplatin exposure. Recent metabolomic profiling of patients with Autism spectrum disorder similarly identified reduced levels of circulating L-tyrosine, supporting an association between altered tyrosine availability and neurological dysfunction [46].

Furthermore, dietary tyrosine supplementation in a mouse model with neurological problems increased hippocampal L-tyrosine levels and attenuated neuronal loss [47]. Based on our LC/MS data on plant metabolites (Table 1), we identified N-acetyl-L-phenylalanine, an acetylated amino acid derivative [48], which may also serve as a potential source of L-phenylalanine bioavailability. As phenylalanine is the direct metabolic precursor of L-tyrosine, AC-EA may support intracellular amino acid availability under stress conditions. 

In parallel, citric acid was identified as a metabolite directly linked to the TCA cycle (Figure 9). Because the TCA cycle is essential for mitochondrial activity, changes in citric acid abundance may reflect alterations in mitochondrial substrate metabolism. Previous work has demonstrated that depletion of neuronal TCA-cycle-related enzymes contributes to axonal ATP deficiency during neuroinflammatory injury, underscoring the importance of TCA cycle maintenance for neuronal energy [49]. Therefore, normalization of citric acid following AC-EA co-treatment may be consistent with mitochondrial status. These metabolic findings coincided with the recovery of MitoTracker fluorescence.

Moreover, AC-EA co-treatment restored intracellular β-alanine, suggesting partial recovery of neuronal amino acid homeostasis. In a recent study, metabolomic profiling showed that β-alanine abundance and β-alanine-associated metabolism were reduced in neurons of an Alzheimer’s disease mouse model, indicating that disruption of β-alanine homeostasis may accompany neuronal metabolic dysfunction [50]. β-alanine is also a key precursor for carnosine biosynthesis, and carnosine plays a major role in antioxidant and intracellular defense [51]. Restoring β-alanine may therefore enhance cellular antioxidant defense capacity. However, because carnosine was not measured in the present study, increased β-alanine cannot be considered direct evidence of enhanced carnosine biosynthesis. This aligns with a previous study demonstrating that carnosine exerts neuroprotective effects against metal-induced neurotoxicity through antioxidant and mitochondrial protective mechanisms, confirming its potential to mitigate oxidative injury [52]. Collectively, these metabolomic changes suggest that AC-EA supports neuronal resilience by promoting metabolic adaptations that replenish neurotransmitter precursor availability and restore intracellular redox balance during cisplatin-induced neurotoxicity.

In silico docking analysis generated hypotheses about potential interactions between putative AC-EA phytocompounds and KEAP1 or aminoacylase-1. Phytocompounds, including daphnoline, rhodexin A, and catechin, showed predicted binding interactions with both proteins, suggesting possible links to redox regulation and amino acid metabolism. These predicted interactions may contribute to modulation of NRF2-associated antioxidant signaling and to the levels of selected intracellular metabolites observed in the present study. 

Although *A. catechu* contains multiple pharmacologically active compounds, long-term areca nut intake is linked to oral cancer and other health issues. Therefore, the therapeutic benefits observed in this study should be interpreted cautiously. Future research should aim to identify the neuroprotective agents responsible for these effects and to determine whether fractions lacking arecoline still provide neuroprotection with fewer toxic risks.

Several limitations should be acknowledged. First, the study was conducted primarily in the HT22 immortalized hippocampal cell line and therefore requires validation in primary neuronal cultures and mammalian models of chemotherapy-induced peripheral neuropathy. Second, although increased pNRF2 expression and GST-4 activation support the involvement of antioxidant signaling, direct confirmation of NRF2 target gene activation was not performed. Third, metabolomic analysis identifies associations rather than causal mechanisms. Fourth, the absence of the AC-EA-only group in the worm experiment prevents distinguishing between cisplatin-specific protection and a general increase in stress resistance.

Our study showed that the AC-EA extract provided significant neuroprotection against cisplatin-induced toxicity by preserving mitochondrial status, mitigating oxidative stress, and activating the NRF2-associated antioxidant response, while maintaining total AKT protein levels. Moreover, our metabolomics analyses revealed that AC-EA stabilized several critical amino acids and energy and redox metabolic networks disrupted by cisplatin exposure. These findings demonstrate the protective effects of AC-EA against cisplatin-induced cellular toxicity in HT22 cells and support further validation in primary sensory neurons and mammalian models of chemotherapy-induced peripheral neuropathy.

## 4. Materials and Methods

### 4.1. Chemicals and Reagents

Dulbecco’s Modified Eagle’s Medium (DMEM) and Bradford reagent were purchased from Sigma-Aldrich Co. (St. Louis, MO, USA). Fetal bovine serum and 0.25% trypsin-EDTA were purchased from Gibco BRL (Life Technologies, Paisley, UK). 3-(4,5-dimethylthiazol-2-yl)-2,5-diphenyltetrazolium bromide (MTT) was obtained from Bio Basic (Markham, ON, Canada). cis-Diamminedichloroplatinum(II) was obtained from TCI Chemicals (Tokyo, Japan). All solvents were of high-performance liquid chromatography (HPLC) grade. Water used in this study was purified using a Milli-Q system from Millipore. All primary antibodies were purchased from Cell Signaling Technology (Beverly, MA, USA) and Abcam (Cambridge, UK). MitoTracker^TM^ Red CM-H_2_TMRos for mitochondrial labeling and H_2_DCFDA fluorescent dye were obtained from Thermo Fisher Scientific (Waltham, MA, USA).

### 4.2. Preparation of AC Extract

*A. catechu* (AC) fruits were collected locally in Surat Thani in 2022 and botanically validated by the Chulalongkorn University herbarium (BCU 016434). The husks were isolated, washed, dried, and pulverized. The resulting AC powder was exhaustively extracted with ethyl acetate using a Soxhlet apparatus. The solvent was removed by rotary evaporation at a maximum temperature of 50 °C. The dried crude extract, designated AC-EA, was reconstituted in DMSO to 100 mg/mL as a stock solution, then stored at −20 °C in the dark. Working concentrations were freshly prepared in culture medium to ensure that the final DMSO concentration did not exceed 0.1%.

### 4.3. Phytochemical Constituent Analysis of AC Extract by LC-MS/MS

The ethyl acetate extract of AC-EA was analyzed by LC-MS/MS to detect bioactive components. Separation was performed on a Thermo Scientific C18 column (AcclaimTM Polar Advantage II, 3 × 150 mm, 3 µm particle size; Thermo Fisher Scientific, Sunnyvale, CA, USA) on an UltiMate 3000 UHPLC system (Dionex Softron GmbH, Germering, Germany). Gradient elution was performed at a flow rate of 0.4 mL/min and a column temperature of 40 °C using H_2_O + 0.1% Formic Acid (A) and 100% ACN (B), with a total run time of 22 min. The injection volume for the sample was 3 µL. The gradient started at 5% B (0–3 min); 80% B (3–10 min); 80% B (10–15 min), and 5% B (15–22 min). High-resolution mass spectrometry was carried out using a MicroTOF QIII Bruker Daltonics instrument (Bruker Daltonik GmbH, Bremen, Germany) ESI-positive ion mode with the following settings: capillary voltage: 4500 V; nebulizer pressure: 2.0 bar; drying gas: 8 L/min at 250 °C. The mass range was 50–1000 *m*/*z*. The accurate mass data of the molecular ions, provided by the TOF analyzer, were processed using Compass Data Analysis software (Bruker Daltonik GmbH). Metabolites from AC-EA were identified using the online tools MetFrag and METLIN [53,54].

### 4.4. Molecular Docking

#### 4.4.1. Protein and Ligand Preparation

The 3D protein structures of the host metabolic targets Kelch-like ECH-associated protein 1 (KEAP1) and aminoacylase-1 were downloaded from the AlphaFold database (https://alphafoldserver.com/, accessed on 22 November 2025). The structures were prepared with polar hydrogens and Gasteiger-Kollman charges using AutoDock Tools (version 1.5.7) and saved as PDBQT receptor files for docking studies. The 3D SDF chemical structures of phytocompounds were downloaded from the PUBCHEM database (https://pubchem.ncbi.nlm.nih.gov/, accessed on 22 November 2025) and converted to PDBQT format after energy minimization with the MMFF94 force field for docking analysis.

#### 4.4.2. Molecular Docking Analysis

Blind docking across the entire protein surface was performed using AutoDock Vina (version 1.5.6) in a shell-script-based automated workflow to elucidate interactions between phytocompounds and the target protein and to shed light on the underlying biochemical processes. This approach facilitated high-throughput docking of multiple phytocompounds against each target, with the grid box and docking parameters. The docked poses of the ligand–protein pairs were ranked by calculated binding free energy (kcal/mol) and visualized in Discovery Studio Visualizer to identify the contributions of different interactions to ligand binding, such as hydrogen bonds and hydrophobic interactions.

### 4.5. Cell Culture and Cell Viability Determination Through MTT Assay

HT22 mouse hippocampal neuronal cells, a kind gift from Professor David Schubert at the Salk Institute (San Diego, CA, USA), and A549 cells (American Type Culture Collection, Manassas, VA, USA ) were maintained in DMEM supplemented with 10% FBS, 1% penicillin-streptomycin, and 2 mM L-glutamine. Cells were cultured at 37 °C in a humidified atmosphere of 5% CO_2_ and subcultured at 70–80% confluence.

Cell viability was assessed using the MTT colorimetric assay, following the protocol [55] with a minor modification. Cells were seeded into 96-well plates (8000 cells/well) at an appropriate density and treated for 24 h with Cisplatin (10 µM) and/or AC-EA extracts at concentrations of 10–100 µg/mL. 10 µL of MTT (5 mg/mL) was added to each well, and the plate was incubated for 4 h at 37 °C. Formazan crystals were dissolved in DMSO, and absorbance was measured at 570 nm using the EnSpire Multimode Plate Reader (PerkinElmer, Waltham, MA, USA). Cell viability was expressed as a percentage of the untreated control.

### 4.6. Intracellular ROS Measurement

Intracellular reactive oxygen species (ROS) levels were quantified using the DCFH-DA fluorescence method as previously described [56] with minor modification. After 24 h treatments, cells were washed with PBS and incubated with DCFH-DA (10 µM) for 30 min at 37 °C in the dark. The excess probe was removed by washing with PBS. Fluorescence intensity was measured at excitation/emission wavelengths of 485/535 nm using the EnSpire Multimode Plate Reader (PerkinElmer, Waltham, MA, USA). Total cellular protein was measured from identically treated parallel wells using the Bradford assay. DCFH-DA fluorescence was normalized to total protein content and expressed as RFU/µg protein.

### 4.7. Mitochondrial Staining and Fluorescence Analysis

Mitochondria-associated fluorescence was evaluated using MitoTracker Red CM-H_2_TMRos (Thermo Fisher Scientific, Waltham, MA, USA), according to the manufacturer’s instructions. For the mitochondrial morphology analysis, HT22 cells were grown on coverslips in 6-well plates. Following the treatments, the cells were stained with 200 nM MitoTracker Red and incubated in the dark for 45 min, then washed and fixed with 4% paraformaldehyde at room temperature for 15 min. Subsequently, cells were permeabilized with 0.5% Triton X-100 for 10 min and counterstained with DAPI. After a final rinse with PBS, stained cells were mounted on a slide and examined using a confocal microscope (Zeiss LSM 800, Carl Zeiss AG, Oberkochen, Germany, Halal Science Center, Chulalongkorn University). MitoTracker fluorescence intensity was quantified using ImageJ software (version 1.54p; National Institutes of Health, Bethesda, MD, USA) and normalized to the control group.

### 4.8. Western Blotting

Protein extraction and Western blot analysis were performed following a previously described protocol [16]. HT22 cells plated in 6-well plates were treated with control, cisplatin 10 µM, AC-EA 10 µg/mL + cisplatin 10 µM, and AC-EA 100 µg/mL + cisplatin 10 µM for 24 h. The cells were lysed using RIPA lysis buffer, incubated on ice for 30 min, then centrifuged at 12,000 × *g* for 15 min at 4 °C, and the supernatant was collected for SDS-PAGE. An equal amount of protein (20 µg) from each treatment was denatured by heating in Laemmli buffer at 95 °C for 10 min. The denatured protein was separated on a 10% SDS polyacrylamide gel (SDS-PAGE) before protein transfer to the PVDF membrane. Membranes were blocked for one hour in 5% BSA (bovine serum albumin) in TBS-T (Tris-buffered saline with 0.1% Tween 20) at room temperature. The membranes were then incubated overnight at 4 °C with the primary antibodies: pH2AX (1:1000), pNRF2 (EP1809Y, Abcam, ab76026; 1:1000), total AKT (1:1000), iNOS (1:1000), and β-actin (1:5000). HRP-conjugated anti-rabbit IgG secondary antibody (1:5000, Cell Signaling Technology, Inc., Danvers, MA, USA) was used. Although NRF2 has a predicted molecular mass of approximately 68 kDa, it exhibits anomalous electrophoretic migration and is commonly detected at approximately 95–110 kDa by SDS-PAGE [57]. HRP signals were detected by enhancing with chemiluminescent reagent (ECL^TM^ Select Western blotting detection reagent: GE Healthcare, Piscataway, NJ, USA) on Chemidoc IQ800 (Cytiva, Marlborough, MA, USA). The intensity of the bands was quantified using ImageJ software.

### 4.9. C. elegans Maintenance and In Vivo Oxidative Stress Assay (Juglone Survival)

The N2 Bristol strain of wild-type *C. elegans* was procured from the Caenorhabditis Genetics Center (CGC) at the University of Minnesota, Minneapolis, MN, USA. They were grown on *Escherichia coli* OP50 (*E. coli* OP50)-coated nematode growth medium (NGM) in a 15 °C incubator. Synchronization of the worms was performed by lysing gravid nematodes in lysis buffer (5 M NaOH and 5% NaOCl) with vigorous vortexing for 6 min. The eggs were harvested by centrifuging at 1800 rpm for 2 min, followed by washing twice with M9 buffer. The eggs were left to hatch overnight at 15 °C to obtain the L1 larvae. The L1 larvae were moved to fresh NGM plates coated with *E. coli* OP50 and grown to the L4 stage before conducting subsequent experiments.

To evaluate the potential of AC-EA to confer resistance against oxidative stress, a survival assay was conducted using the pro-oxidant juglone (5-hydroxy-1,4-naphthoquinone) in wild-type (N2) *C. elegans*. Since cisplatin toxicity is driven by ROS generation, adding juglone provides a controlled model of acute oxidative collapse to test the extract’s protective efficacy. A 24 h pretreatment window was considered to allow the activation of endogenous antioxidant defenses prior to the acute oxidative stress [58].

Synchronized worms were transferred to 24-well plates (approximately 10 nematodes per well) containing 400 µL of M9 buffer supplemented with *E. coli* OP50 as a food source. To establish the pretreatment conditions, the worms were divided into control, cisplatin alone (10 µM), and co-treatment with AC-EA and cisplatin groups. Following a 24 h incubation at 20 °C, worms were exposed to freshly prepared juglone (50 µM per well) at the beginning of the assay. Juglone was administered once and was not replenished during the subsequent survival monitoring period. Nematode survival was scored at every 48 h interval. The animals were recorded as dead when they did not respond to mechanical stimuli. Worm survival was analyzed in GraphPad Prism 9, and the log-rank test was used to assess statistical differences between experimental treatments. All assays on *C. elegans* were performed in accordance with the institutional biosafety guidelines.

### 4.10. Analysis of Antioxidant Gene Expression Using Transgenic C. elegans Reporters

Synchronized L1 larvae of the CL2166 strain (GST-4 reporter strain) were treated with the test compounds in M9 buffer containing E. coli OP50 and incubated for 72 h until reaching the adult stage. The worms were washed three times with M9 buffer. Approximately 30 worms were transferred onto slides, and 10 mM NaN_3_ was added to paralyze them. Fluorescence was measured using a Zeiss LSM 800 confocal laser scanning microscope at the Halal Science Center, Chulalongkorn University, under standard settings. Results were expressed as relative fluorescence intensity.

### 4.11. Statistical Analysis

Data are presented as the mean ± standard deviation (SD). The number of replicates are specified in the corresponding figure legends and the experimental section. Statistical analyses were performed using GraphPad Prism 9 (GraphPad Software, Inc., San Diego, CA, USA). Group differences were evaluated using either an unpaired *t*-test or one-way ANOVA with Dunnett’s post hoc test. A *p*-value < 0.05 was considered statistically significant.

### 4.12. Metabolomics Analysis

#### 4.12.1. Sample Preparation

HT22 cells were seeded at 2 × 10^6^ cells per well in 6-well plates. Cells were assigned to three experimental groups: Control, Cisplatin (10 µM), and Co-treatment (AC-EA 100 µg/mL plus Cisplatin 10 µM). To extract intracellular metabolites, cells were first washed with ice-cold PBS to remove residual culture medium. Metabolic activity was immediately quenched by adding 80% cold methanol, and the mixture was homogenized with an ultrasonicator at 40% for 2 min. The lysate was centrifuged at 12,000 rpm for 15 min at 4 °C to remove debris. Supernatants were transferred to a 1.5 mL Eppendorf tube and dried under nitrogen, as described earlier [59].

The dried samples were stored at −80 °C until LC-QTOF-MS/MS analysis. They were reconstituted with 500 µL of mobile phase A, filtered through a 0.22 µm membrane filter, and transferred to amber glass vials. Six replicates per treatment group were analyzed, and 50 µL of each sample was pooled in a 2 mL Eppendorf tube to create a quality control (QC) sample.

#### 4.12.2. Data Acquisition

Untargeted metabolomic profiling was performed on a Dionex UltiMate 3000 UHPLC system (Thermo Fisher Scientific, Waltham, MA, USA) coupled to a QTOF Impact II mass spectrometer (Bruker Daltonics, Bremen, Germany). Chromatographic separation was achieved on a C18 column (2.1 × 100 mm, 1.9 µm particle size; Thermo Fisher Scientific, Sunnyvale, CA, USA) maintained at 40 °C, with the autosampler temperature set to 7 °C. The mobile phase consisted of 0.1% formic acid in water (Solvent A) and 0.1% formic acid in acetonitrile (Solvent B), delivered at a flow rate of 0.3 mL/min. The multistep gradient elution was programmed as follows: 0% to 99% B over 15 min, held at 99% B for 5 min, rapidly decreased to 1% B between 20 and 20.1 min, and re-equilibrated at 1% B for 5 min, resulting in a total run time of 25 min.

Mass spectrometry data were acquired in both positive and negative electrospray ionization (ESI) modes over an *m*/*z* range of 50–1200. Nitrogen served as both the nebulizer and collision gas, with collision energies set to 20.0 eV and 10.0 eV, respectively. Ion source parameters were optimized as follows: dry gas temperature of 250 °C, dry gas flow rate of 8.0 L/min, and capillary voltages of 3800 V for positive mode and 2500 V for negative mode.

#### 4.12.3. Data Processing and Statistical Analysis

Raw LC-MS/MS datasets were converted to the Analysis Base File (ABF) format with the Reifycs Analysis Base File Converter (Reifycs Inc., Tokyo, Japan). Subsequent data processing, including spectral deconvolution, peak detection, alignment, and metabolite annotation, was performed with MS-DIAL 5.1. Putative compound identification was achieved by matching spectral features against multiple public databases, including MassBank, GNPS, LipidBlast 2022, and HMDB.

Statistical analysis and visualization were conducted using MetaboAnalyst 6.0. To ensure data quality, variables were filtered using an interquartile range (relative standard deviation of 20%) and a mean intensity threshold of 5%. The resulting dataset was normalized using a log transformation and Pareto scaling. To discriminate metabolic profiles between groups, Partial Least Squares-Discriminant Analysis (PLS-DA) was employed. Potential biomarkers were selected based on Variable Importance in Projection (VIP) scores and statistical significance (*p* < 0.05). Finally, pathway analysis was performed to characterize the specific metabolic networks modulated by AC-EA treatment in HT22 cells.

## Figures and Tables

**Table 1:** Putative annotated phytocompounds detected in AC-EA by LC-MS/MS analysis of AC-EA.

No.	Phytocompounds in AC-EA	Retention Time (min)	*m*/*z* Ratio	Molecular Formula
1	Arecoline	1.8	156.1011	C_8_H_13_NO_2_
2	Hydroxyhydroquinone	8.4	127.0375	C_6_H_6_O_3_
3	Salicylic acid	8.6	139.0382	C_7_H_6_O_3_
4	(±) Catechin	8.6	291.0877	C_15_H_14_O_6_
5	N-Acetyl-L-phenylalanine	9.4	208.097	C_11_H_13_NO_3_
6	Phytosphingosine	9.5	318.3024	C_18_H_39_NO_3_
7	Shanzhiside	10.4	393.1368	C_16_H_24_O_11_
8	Burseran	11.6	387.1844	C_22_H_26_O_6_
9	Magnoshinin	12.0	415.2167	C_24_H_30_O_6_
10	Indospicine	12.5	174.1273	C_7_H_15_N_3_O_2_
11	3-Butylidene-7-hydroxyphthalide	13.5	205.0869	C_12_H_12_O_3_
12	Echinenone/Myxoxanthin	13.7	573.4039	C_40_H_54_O
13	Rhodexin A	14.7	559.2987	C_29_H_44_O_9_
14	Montanol	14.8	353.2715	C_21_H_36_O_4_
15	Daphnoline	15.4	581.2473	C_35_H_36_N_2_O_6_
16	Stearic acid	16.5	285.28	C_18_H_36_O_2_
17	cis-gondoic acid	17.0	311.296	C_20_H_38_O_2_
18	13-Docosenoic acid	17.0	339.3268	C_22_H_42_O_2_

**Table 2:** Selected nominally enriched pathway analysis of identified metabolites.

	Pathway Name	Match Status	*p*	−log(*p*)	Holm *p*	FDR	Impact
1	Phenylalanine, tyrosine and tryptophan biosynthesis	3/4	0.037505	1.4259	1	0.14854	1
2	Phenylalanine metabolism	3/10	0.037505	1.4259	1	0.14854	0.61904
3	β-Alanine metabolism	2/21	0.004832	2.3159	0.21742	0.060417	0.39925

Raw *p*-values are shown together with Holm-adjusted *p*-values and FDR values. None of the selected pathways remained significant after correction for multiple comparisons.

## Data Availability

The original contributions presented in this study are included in the article/Supplementary Materials. Further inquiries can be directed to the corresponding authors.

## Supplementary Materials

The following supporting information can be downloaded at: https://www.mdpi.com/article/10.3390/ijms27157004/s1.

## Abbreviations

The following abbreviations are used in this manuscript:

AC-EA	*Areca catechu* ethyl acetate extract
BSA	Bovine serum albumin
DCFH-DA	2′,7′-dichlorodihydrofluorescein diacetate
DMEM	Dulbecco’s Modified Eagle’s Medium
DMSO	Dimethyl sulfoxide
DRG	Dorsal root ganglia
DSB	Double-strand break
ESI	Electrospray ionization
FBS	Fetal bovine serum
GST-4	Glutathione S-transferase 4
HMDB	Human Metabolome Database
HPLC	High-performance liquid chromatography
HRP	Horseradish peroxidase
IL-1β	Interleukin-1 beta
iNOS	Inducible nitric oxide synthase
KEAP1	Kelch-like ECH-associated protein 1
LC-MS/MS	Liquid chromatography-tandem mass spectrometry
MTT	3-(4,5-dimethylthiazol-2-yl)-2,5-diphenyltetrazolium bromide
NGM	Nematode growth media
NRF2	Nuclear factor erythroid 2-related factor 2
OPLS-DA	Orthogonal Partial Least Squares-Discriminant Analysis
PBS	Phosphate-buffered saline
PCA	Principal Component Analysis
PDBQT	Protein Data Bank, Partial Charge (Q), and Atom Type (T)
PLS-DA	Partial Least Squares-Discriminant Analysis
PVDF	Polyvinylidene fluoride
RIPA	Radioimmunoprecipitation assay
ROS	Reactive oxygen species
SDS-PAGE	Sodium dodecyl sulfate-polyacrylamide gel electrophoresis
TCA	Citric acid cycle (Tricarboxylic acid cycle)
TOF	Time-of-flight
VIP	Variable Importance in Projection
γH2AX	Gamma-H2A histone family member X
