# Supplementary material for: Multi-Targeted Neuroprotection by Areca catechu Against Cisplatin-Induced Neurotoxicity: Cellular, Caenorhabditis elegans, and Metabolomic Evidence
Source: Int J Mol Sci. 2026 Aug 4;27(15):7004. doi: 10.3390/ijms27157004 (PMC13467248; doi:10.3390/ijms27157004)
Supplement: Supplementary file 1 [file ijms-27-07004-s001.zip › Supplementary Figures S1 and S2.pdf]

Acquisition Parameter-	Instrument micrOTOF-Q III 8228888.20450	
Source Type- ESI	Ion Polarity-Positive	Set Nebulizer-2.0 Bar
Focus Not-active	Set Capillary-4500 V	Set Dry Heater-300 °C
Scan Begin-50 m/z	Set End Plate Offset -500 V	Set Dry Gas-8.0 l/min
Scan End-1000 m/z	Set Collision Cell RF -200.0 Vpp	Set Divert Valve-Waste

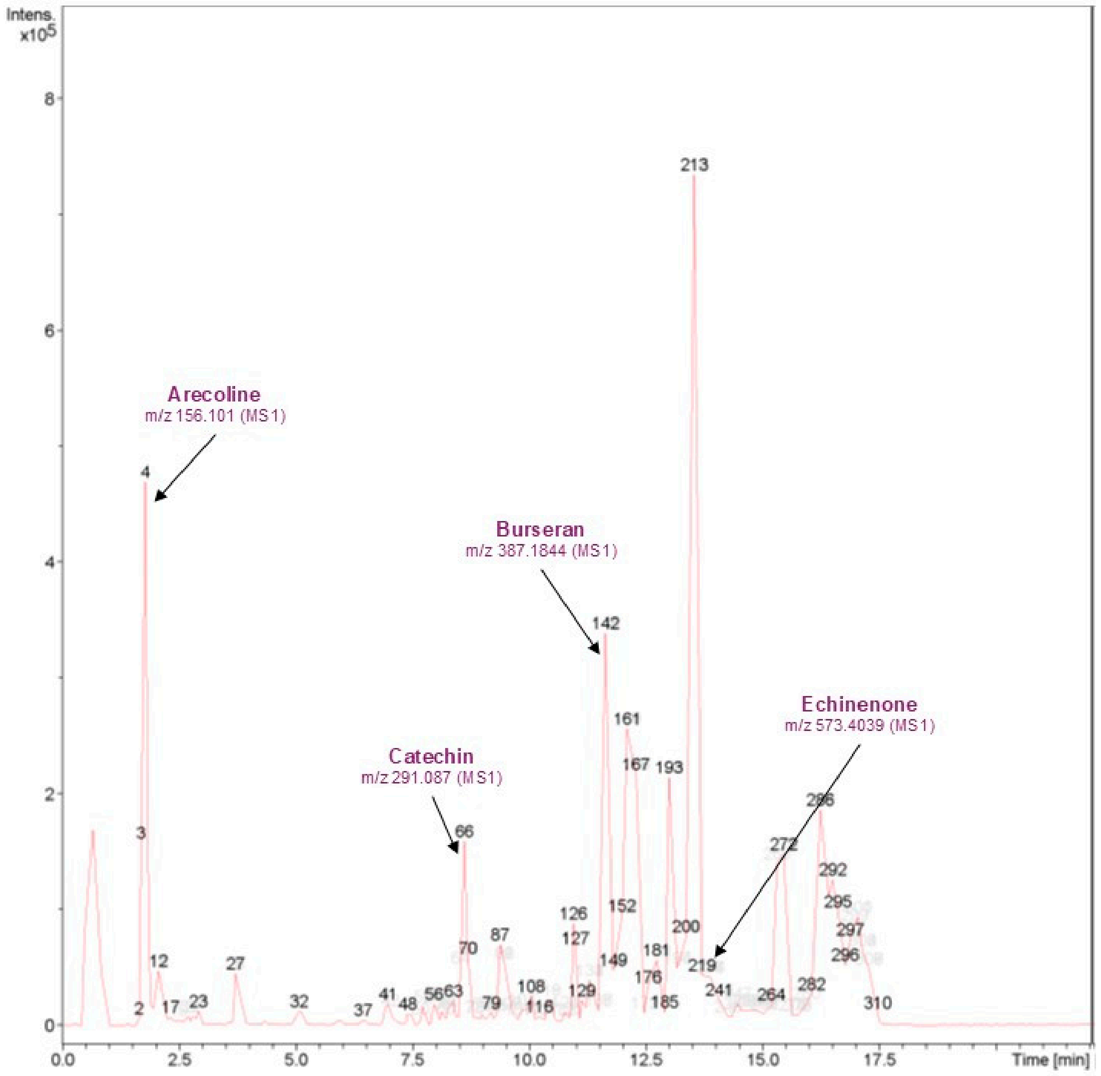

Supplementary Figure S1: LC–MS chromatographic profile of AC-EA

A

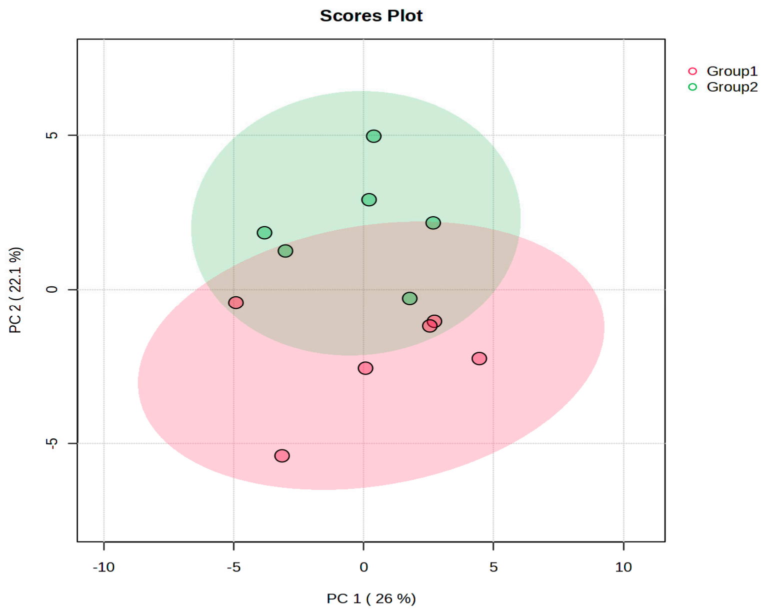

B

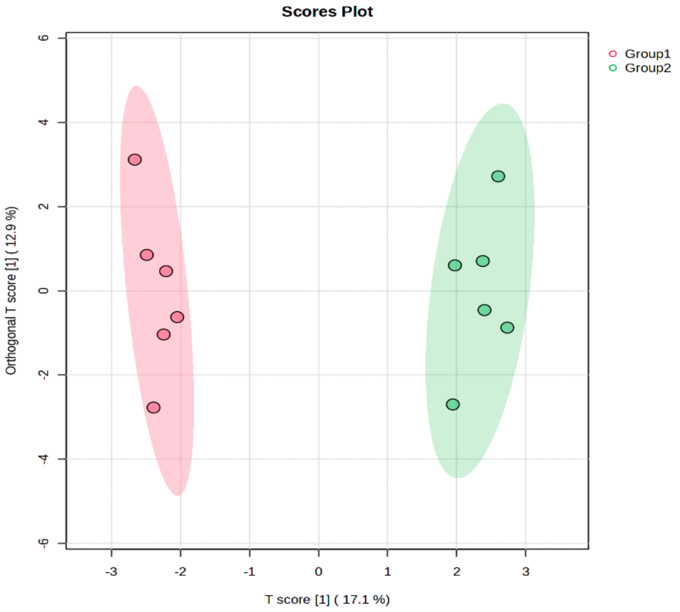

Supplementary Figure S2: PCA (A) and OPLS-DA (B) score plots of metabolic profile differences between cisplatin and AC-EA co-treated HT22 cells.
