# Supplementary material for: Multi-Targeted Neuroprotection by Areca catechu Against Cisplatin-Induced Neurotoxicity: Cellular, Caenorhabditis elegans, and Metabolomic Evidence
Source: Int J Mol Sci. 2026 Aug 4;27(15):7004. doi: 10.3390/ijms27157004 (PMC13467248; doi:10.3390/ijms27157004)
Supplement: Supplementary file 1 [file ijms-27-07004-s001.zip › Supplementary Table S1.pdf]

Supplementary Table S1: Molecular docking scores of the studied compounds in interaction with the KEAP1 and Aminoacylase-1 protein

Docking score in interaction with KEAP1

Sr. No.	Compound	Docking Score (kcal/mol)
1	Rhodexin A	-10.793
2	Daphnoline	-9.964
3	(±)-Catechin	-9.201
4	Shanzhiside	-8.158
5	N-Acetyl-L-phenylalanine	-6.841
6	Senkyunolide B	-6.839
7	Montanol	-6.53
8	Echinenone	-6.458
9	Magnoshinin	-6.118
10	Salicylic Acid	-5.685
11	Indospicine	-5.652
12	Hydroxyhydroquinone	-5.596
13	Burseran	-5.403
14	Arecoline	-5.307
15	13-Docosenoic Acid	-5.055
16	Phytosphingosine	-4.948
17	Cis-gondoic Acid	-4.684
18	Stearic Acid	-3.22

Docking score in interaction with Aminoacylase1

Sr. No.	Phytocompounds	Docking Scores (kcal/mol)
1	Daphnoline	-7.911
2	Rhodexin A	-7.629
3	Echinenone	-6.951
4	(±)-Catechin	-6.612
5	Shanzhiside	-6.446
6	Burseran	-6.137
7	N-Acetyl-L-phenylalanine	-6.038
8	Salicylic Acid	-5.794
9	Senkyunolide B	-5.653
10	Montanol	-5.491
11	13-Docosenoic Acid	-5.457
12	Hydroxyhydroquinone	-5.126
13	Stearic Acid	-4.532
14	Indospicine	-4.315
15	Arecoline	-4.28
16	Cis-gondoic Acid	-3.777
17	Docosenoicacid	-3.373
18	Phytosphingosine	-3.359
