# Supplementary material for: Multi-Targeted Neuroprotection by Areca catechu Against Cisplatin-Induced Neurotoxicity: Cellular, Caenorhabditis elegans, and Metabolomic Evidence
Source: Int J Mol Sci. 2026 Aug 4;27(15):7004. doi: 10.3390/ijms27157004 (PMC13467248; doi:10.3390/ijms27157004)
Supplement: Supplementary file 1 [file ijms-27-07004-s001.zip › Supplementary Table S4.pdf]

**Supplementary Table S4: Metabolic pathway enrichment analysis of the identified metabolites ranked by p-value significance.**

| | Pathway Name | Match Status | p | -log(p) | Holm p | FDR | Impact |
|---|---|---|---|---|---|---|---|
| 1 | β-Alanine metabolism | 2/21 | 0.0048315 | 2.3159 | 0.21742 | 0.060417 | 0.39925 |
| 2 | Pantothenate and CoA biosynthesis | 2/20 | 0.0048315 | 2.3159 | 0.21742 | 0.060417 | 0.04762 |
| 3 | Valine, leucine and isoleucine degradation | 2/40 | 0.0052536 | 2.2795 | 0.22591 | 0.060417 | 0.0 |
| 4 | Pyrimidine metabolism | 10/39 | 0.008534 | 2.0688 | 0.35843 | 0.078513 | 0.3438 |
| 5 | Ascorbate and aldarate metabolism | 2/10 | 0.013514 | 1.8692 | 0.55409 | 0.10361 | 0.16216 |
| 6 | Glyoxylate and dicarboxylate metabolism | 2/32 | 0.035145 | 1.4541 | 1.0 | 0.14854 | 0.16333 |
| 7 | Ubiquinone and other terpenoid-quinone biosynthesis | 1/19 | 0.035289 | 1.4524 | 1.0 | 0.14854 | 0.0 |
| 8 | Phenylalanine, tyrosine and tryptophan biosynthesis | 3/4 | 0.037505 | 1.4259 | 1.0 | 0.14854 | 1.0 |
| 9 | Phenylalanine metabolism | 3/10 | 0.037505 | 1.4259 | 1.0 | 0.14854 | 0.61904 |
| 10 | Tyrosine metabolism | 4/42 | 0.039808 | 1.4 | 1.0 | 0.14854 | 0.20072 |
